# Differences in gait characteristics between total hip, knee, and ankle arthroplasty patients: a six-month postoperative comparison

**DOI:** 10.1186/1471-2474-14-176

**Published:** 2013-06-03

**Authors:** Nicola C Casartelli, Julia F Item-Glatthorn, Mario Bizzini, Michael Leunig, Nicola A Maffiuletti

**Affiliations:** 1 Neuromuscular Research Laboratory, Schulthess Clinic, Zurich, Switzerland; 2 INSERM U1093 - Cognition, Action and Sensory Plasticity, University of Burgundy, Dijon, France; 3 Hip Service, Schulthess Clinic, Zurich, Switzerland

**Keywords:** Gait, Arthroplasty, Hip, Knee, Ankle

## Abstract

**Background:**

The recovery of gait ability is one of the primary goals for patients following total arthroplasty of lower-limb joints. The aim of this study was to objectively compare gait differences of patients after unilateral total hip arthroplasty (THA), total knee arthroplasty (TKA) and total ankle arthroplasty (TAA) with a group of healthy controls.

**Methods:**

A total of 26 TAA, 26 TKA and 26 THA patients with a mean (± SD) age of 64 (± 9) years were evaluated six months after surgery and compared with 26 matched healthy controls. Subjects were asked to walk at self-selected normal and fast speeds on a validated pressure mat. The following spatiotemporal gait parameters were measured: walking velocity, cadence, single-limb support (SLS) time, double-limb support (DLS) time, stance time, step length and step width.

**Results:**

TAA and TKA patients walked slower than controls at normal (*p*<0.05) and fast speeds (*p*<0.01). The involved side of TAA and TKA patients showed shorter SLS compared to controls at both normal and fast speeds (*p*<0.01). Regardless of walking speed, the uninvolved side of TAA and TKA patients demonstrated longer stance time and shorter step length than controls (*p*<0.01). TAA patients showed shorter SLS of the involved side, longer stance time and shorter step length of the uninvolved side compared to the contralateral side at both normal and fast speeds (*p*<0.001).

**Conclusions:**

Gait disability after unilateral lower-limb joint arthroplasty was more marked for distal than for proximal joints at six months after surgery, with a proximal-to-distal progression in the impairment (TAA>TKA>THA). THA patients demonstrated no gait differences compared with controls. In contrast, TAA and TKA patients still demonstrated gait differences compared to controls, with slower walking velocity and reduced SLS in the involved limb. In addition, TAA patients presented marked side-to-side asymmetries in gait characteristics.

## Background

Total hip arthroplasty (THA) and total knee arthroplasty (TKA) are currently the most effective surgical interventions for the treatment of end-stage hip and knee arthritis [1]. They have been proven to be successful in reducing joint pain and stiffness as well as in improving physical function, with THA patients usually demonstrating earlier and more pronounced improvements in comparison to TKA patients [2]. Total ankle arthroplasty (TAA) is not the standard surgical procedure for the treatment of end-stage ankle arthritis but rather an increasingly valid alternative to ankle arthrodesis [3]. After the failure of first-generation ankle prostheses due to high postoperative complication rates [4], modern three-component ankle implants seem to provide intermediate- and long-term clinical outcomes comparable to those of ankle arthrodesis [5]. Since TAA preserves ankle joint motion better than ankle arthrodesis, TAA would reduce the risk of developing arthritis in adjacent foot joints and minimize the deterioration in walking function [6].

The recovery of gait function is one of the primary goals for patients, surgeons and physical therapists following lower-limb total joint arthroplasty. Indeed, gait ability represents the simplest but most necessary physical activity in everyday life [7]. Recovery of gait function not only allows the patients to regain independence in a large number of daily activities (e.g., household works, shopping), but also to regain an active lifestyle which is essential for restoring lower-limb muscle function after surgery. Several studies have already investigated gait function of patients after THA [8-10], TKA [11-13] and TAA [6,14-16]. To our knowledge, however, the gait characteristics of these three groups of patients have never been compared in a systematic fashion. This would provide valuable information about differences in objective functional recovery between patients who have had a primary total arthroplasty of a main weight-bearing joint. Therefore, the aim of this study was to compare spatiotemporal gait parameters between matched healthy controls and THA, TKA and TAA patients six months after surgery. It was hypothesized that patients following THA, TKA and TAA would demonstrate gait disturbances compared to healthy controls, with THA and TAA patients presenting the least and greatest gait impairment, respectively.

## Methods

### Subjects

A total of 26 TAA patients, 26 TKA patients, 26 THA patients, and 26 healthy controls were included in the study (Table 1). The sample size was determined based on the walking velocity meaningful change found for orthopedic patients (i.e., 10 cm/s) [17]. Power analysis indicated that a minimum sample size of 23 subjects in each group was required to detect significant walking velocity differences between patients and controls (effect size = 1.0, α = 0.05, power = 0.90) [18]. The four groups were matched for gender, age, body mass and height. Patients were evaluated at a mean of 6.0 (± 0.7) months after surgery, and after conclusion of physical therapy. They all underwent primary unilateral total joint arthroplasty in the same orthopedic institute with an indication of end-stage osteoarthritis or post-traumatic arthritis (Table 1). Patients with rheumatoid arthritis were not included in the study. Patients and healthy controls had had no prior surgery to the lower limbs (except joint arthroplasty in patients), and symptoms or signs referable to other overt cardiorespiratory, orthopedic, neurological or general diseases, which could have negatively influenced walking function. The study was conducted according to the principles expressed in the declaration of Helsinki, and the protocol was approved by the local Ethics Committee. All the subjects signed a written informed consent before participating in the study.

**Table 1:** Subject characteristics

	**TAA**	**TKA**	**THA**	**Controls**	***p *value**
	***N*=26**	***N*=26**	***N*=26**	***N*=26**	
*Demographic*/*anthropometric data*					
Gender (women/men)	16/10	16/10	16/10	16/10	-
Age (years)	63 ± 10	65 ± 8	65 ± 8	62 ± 10	0.61
Body mass (kg)	80 ± 17	80 ± 18	75 ± 17	74 ± 14	0.19
Height (cm)	168 ± 8	169 ± 10	167 ± 9	168 ± 9	0.78
*Indication for surgery*					
Osteoarthritis	*N*=8 (31%)	*N*=18 (69%)	*N*=26 (100%)	-	-
Post-traumatic arthritis	*N*=18 (69%)	*N*=8 (31%)	*N*=0 (0%)	-	-
	• 4 unspecified ankle fractures	• 4 tibia fractures			
	• 3 malleolar fractures	• 2 patella fractures			
	• 3 bimalleolar fractures	• 1 femur fracture			
	• 3 severe ankle sprains	• 1 meniscus tear			
	• 2 talus fractures				
	• 1 trimalleolar fracture				
	• 1 distal tibia fracture				
	• 1 pilon tibial fracture				
*Surgery*-*test interval* (*months*)	5.9 ± 0.6	5.9 ± 0.8	6.2 ± 0.4	-	0.14

*TAA*, Total ankle arthroplasty; *TKA*, Total knee arthroplasty; *THA*, Total hip arthroplasty.

TAA patients were operated on between December 2008 and June 2010 by three experienced foot and ankle surgeons using the anterior surgical approach [3]. All patients received the same unconstrained three-component mobile-bearing ankle prosthesis (Mobility™, DePuy International, Leeds, UK). TKA patients were operated on between April 2006 and March 2010 by four experienced knee surgeons, using a standard medial parapatellar approach [19]. In two cases, a patellar replacement was performed. All TKA patients received the same constrained three-component mobile-bearing knee prosthesis (Innex UCOR™, Zimmer, Warsaw, IN, USA). THAs were executed between March 2007 and February 2010. A total of 14 THA patients were operated on by two experienced hip surgeons using the posterior approach [20]. Twelve of them received an Allofit™ acetabular cup with a CLS™ femoral stem (Zimmer), one an Allofit™ acetabular cup with a Weber™ femoral stem (Zimmer), and one a R3™ acetabular cup with a Polar™ femoral stem (Smith & Nephew, Andover, MA, USA). The other 12 THA patients were operated on by one experienced hip surgeon using the anterior surgical approach [21]. Six of them received an EP-Fit™ acetabular cup with a Polar™ femoral stem (Smith & Nephew), three an Allofit™ acetabular cup with a ML-Taper™ femoral stem (Zimmer), and three an Allofit™ acetabular cup with a Weber™ femoral stem. THA patients operated on with anterior and posterior approaches were considered together since they have similar gait characteristics at six months after surgery [18].

After surgery, all the patients received the same postoperative medical care and were advised to complete individual physical therapy sessions supervised by one therapist (usually one to four cycles of nine sessions). Even if rehabilitation guidelines were given to all patients, physical therapy was not standardized in any patient group. Rehabilitation guidelines for THA and TKA patients included weight-bearing with crutches as tolerated, range of motion, balance and strengthening exercises to start after discharge. In contrast, TAA patients wore a cast for the first two to three weeks postoperatively. Afterwards, they were asked to wear a removable walker boot and started physical therapy, which included partial weight-bearing with crutches as well as passive and active ankle mobilization. At six weeks after TAA, they started full weight-bearing in the removable walker boot, balance and strengthening exercises. For all patient groups, follow-up visits at six months after surgery included clinical examination performed by the surgeon, and gait analysis performed in our research laboratory.

### Procedures and outcomes

Spatiotemporal gait parameters were quantified with a validated electronic walkway designed for gait analysis in clinical settings (GAITRite, CIR Systems Inc., Clifton, NJ, USA) [22,23]. The walkway is 823-cm long and has a sensor area of 732 × 61 cm. Pressure sensors are arranged in a grid pattern with a spatial resolution of 1.27 cm. They are activated by mechanical pressure, and the data are sampled at a frequency of 80 Hz. Subjects wore flat-soled shoes and were instructed to walk at two different self-selected speeds: normal (“walk with a pace that you would use in everyday life”) then fast (“walk as fast as you can without running”) [24]. At each speed, one familiarization trial always preceded the three experimental trials. Each trial started 2 m before and ended 2 m after the mat, in order to maintain constant gait patterns. The rest interval between trials was 60 s.

Data were collected by a series of on-board processors, transferred to a personal computer by way of an interface cable, and transformed into footfall patterns using the dedicated software (GAITRite Gold, Version 3.2b, CIR System Inc., Clifton, NJ, USA). The following spatiotemporal gait parameters were retained:

– walking velocity in cm/s;

– cadence in steps/min;

– single-limb support (SLS) time as a percentage of gait cycle;

– double-limb support (DLS) time as a percentage of gait cycle;

– stance time as a percentage of gait cycle;

– step length in cm;

– step width in cm.

At each speed, data of the three experimental trials were averaged together and gait parameters were evaluated separately by side (except for velocity and cadence). Patients had the two sides defined as involved (i.e., operated side) and uninvolved (i.e., non-operated side). Controls had the two sides randomly allocated as involved and uninvolved (unrestricted randomization, computer random number generator).

### Statistics

Normal distribution of the data was assessed with Shapiro-Wilk tests. One-way ANOVAs were used to compare the means between groups (THA, TKA, TAA, controls) for demographic/anthropometric characteristics, surgery-test interval, walking velocity and cadence. Two-way ANCOVAs (main effects: group and side, covariate: walking velocity) were used to compare the means between groups (THA, TKA, TAA, controls) and sides (involved, uninvolved) for SLS time, DLS time, stance time, step length and step width. Walking velocity was used as the covariate for statistical comparisons because many spatiotemporal gait parameters are velocity-dependent [25]. Tukey’s HSD tests were used for *post*-*hoc* pairwise comparisons of the means. Percent side-to-side asymmetries were calculated as (100 × (mean uninvolved side - mean involved side)/mean uninvolved side) for SLS and stance time, and as (100 × (mean involved side - mean uninvolved side)/mean involved side) for step length. Average asymmetries at normal and fast speeds were reported. Statistical analyses were performed with Statistica 7.0 (StatSoft Inc., Tulsa, OK, USA). The significance level was set at *p*<0.05.

## Results

The results of one-way ANOVAs and two-way ANCOVAs are presented in Table 1 and Table 2.

**Table 2:** **Results of one**-**way ANOVAs and two**-**way ANCOVAs** (**main effects and interactions**)

	**Speed**	**Group effect**	**Side effect**	**Group by side**
Walking velocity	Normal	F=12.38 (*p*<0.001)	-	-
	Fast	F=11.85 (*p*<0.001)	-	-
Cadence	Normal	F=5.60 (*p*=0.001)	-	-
	Fast	F=4.05 (*p*=0.009)	-	-
SLS	Normal	F=2.43 (*p*=0.069)	F=4.75 (*p*=0.032)	F=8.37 (*p*<0.001)
	Fast	F=1.41 (*p*=0.243)	F=3.20 (*p*=0.077)	F=13.62 (*p*<0.001)
DLS	Normal	F=2.50 (*p*=0.064)	F=0.11 (*p*=0.744)	F=0.73 (*p*=0.534)
	Fast	F=1.87 (*p*=0.139)	F=2.94 (*p*=0.089)	F=0.34 (*p*=0.794)
Stance time	Normal	F=2.45 (*p*=0.068)	F=7.66 (*p*=0.007)	F=7.59 (*p*<0.001)
	Fast	F=1.37 (*p*=0.255)	F=4.88 (*p*=0.029)	F=13.99 (*p*<0.001)
Step length	Normal	F=0.63 (*p*=0.595)	F=8.28 (*p*=0.005)	F=7.19 (*p*<0.001)
	Fast	F=0.14 (*p*=0.937)	F=4.98 (*p*=0.028)	F=6.49 (*p*<0.001)
Step width	Normal	F=1.79 (*p*=0.153)	F=2.41 (*p*=0.124)	F=1.48 (*p*=0.226)
	Fast	F=1.55 (*p*=0.207)	F=0.32 (*p*=0.573)	F=1.25 (*p*=0.297)

*SLS*, Single-limb support; *DLS*, Double-limb support.

**Figure 1:**
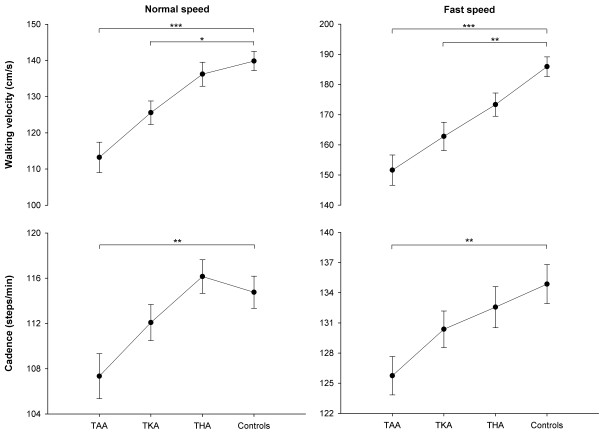
**Walking velocity and cadence of TAA**, **TKA**, **and THA patients and healthy controls.** Circles and error bars represent means and standard errors of the means. *, ** and *** indicate differences between patients and controls at *p*<0.05, *p*<0.01 and *p*<0.001, respectively. TAA: total ankle arthroplasty, TKA: total knee arthroplasty, THA: total hip arthroplasty.

TAA and TKA patients walked slower than controls at both normal (*p*<0.05) and fast speeds (*p*<0.01; Figure 1). Moreover, TAA patients walked with lower cadence compared with controls at both speeds (*p*<0.01).

**Figure 2:**
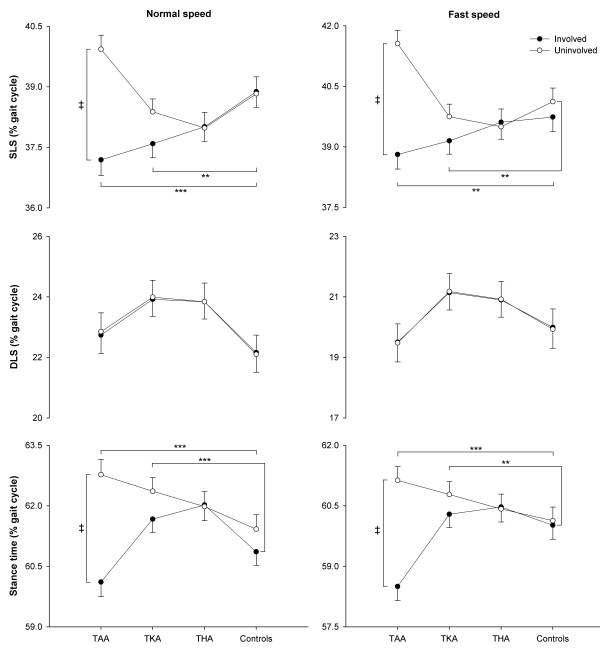
**SLS, DLS and stance time of TAA, TKA**, **and THA patients and healthy controls.** Circles and error bars represent means and standard errors of the means. ** and *** indicate differences between patients and controls at *p*<0.01 and *p*<0.001, respectively. ‡ indicates side-to-side differences in TAA patients at *p*<0.001. SLS: single-limb support, DLS: double-limb support, TAA: total ankle arthroplasty, TKA: total knee arthroplasty, THA: total hip arthroplasty.

At both speeds, the involved side of TAA patients had shorter SLS than controls (*p*<0.01; Figure 2). At normal speed, the involved side of TKA patients had shorter SLS than both sides of controls (*p*<0.01); at fast speed, the involved side of TKA patients had shorter SLS than the uninvolved side of controls (*p*<0.01). In addition, for TAA patients the involved SLS was shorter than the uninvolved one at both speeds (*p*<0.001), with a mean side-to-side asymmetry of 7%. No differences between groups or sides were observed for DLS at any walking speed. At both speeds, the uninvolved side of TAA patients had longer stance time than both sides of controls (*p*<0.001; Figure 2), and the uninvolved side of TKA patients had longer stance time than the involved side of controls (*p*<0.01). In addition, for TAA patients the uninvolved stance time was longer than the involved one at both speeds (*p*<0.001), with a mean side-to-side asymmetry of 4%.

**Figure 3:**
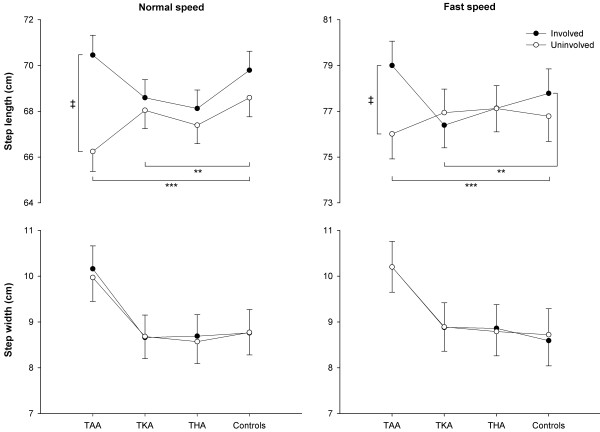
**Step length and step width of TAA, TKA, and THA patients and healthy controls.** Circles and error bars represent means and standard errors of the means. ** and *** indicate differences between patients and controls at *p*<0.01 and *p*<0.001, respectively. ‡ indicates side-to-side differences in TAA patients at *p*<0.001. TAA: total ankle arthroplasty, TKA: total knee arthroplasty, THA: total hip arthroplasty.

At both speeds, the uninvolved side of TAA patients had a shorter step length than controls (*p*<0.001; Figure 3). At normal speed, the uninvolved side of TKA patients had a shorter step length than both sides of controls (*p*<0.01); at fast speed, the uninvolved side of TKA patients had shorter step length than the involved side of controls (*p*<0.01). In addition, for TAA patients the uninvolved step length was shorter than the involved one at both speeds (*p*<0.001), with a mean side-to-side asymmetry of 5%. No differences between groups and sides were observed for step width at any walking speed.

For all parameters, THA patients did not show any significant difference compared with controls at both speeds.

## Discussion

The main findings of this comparison of gait characteristics among lower-limb joint arthroplasty patients were the following: (a) TKA and TAA patients walked at a slower velocity compared to matched healthy controls; (b) TKA and TAA patients had shorter SLS in the involved limb, and longer stance time and shorter step length in the uninvolved limb compared with controls; (c) TAA patients presented considerable side-to-side asymmetries for temporal (SLS, stance time) and spatial (step length) parameters; (d) THA patients did not present any impairment in gait characteristics; (e) between-group differences in walking variables were comparable at normal and fast speeds.

To our knowledge, this is the first study that objectively compared spatiotemporal gait parameters after total arthroplasty of the main weight-bearing joints (hip, knee, and ankle). Our results provide valuable information to orthopedic surgeons and physical therapists about differences between patient subgroups in gait function six months after surgery. In addition, gait parameters were evaluated bilaterally in this study, which allowed the quantification of potential side-to-side asymmetries resulting from compensatory mechanisms adopted by patients to unload the operated joint [26]. These asymmetries may eventually constitute a risk factor for the onset and/or progression of osteoarthritis in the contralateral weight-bearing joints [27]. One obvious limitation of the present study is that gait function of patients was not assessed preoperatively. Therefore, pre- to postoperative changes in gait characteristics cannot be appraised. However, aim of the study was to evaluate the between-group differences in gait restoration at a specific time point after surgery (six months postoperatively), when most of patients have concluded supervised physical therapy. Another limitation of the study is that physical therapy was neither standardized nor quantified. It is however supposed that the potential effects of physical therapy on between-group differences were minimized by the within-group variability of physical therapy amount and intensity.

Walking velocity and SLS were the gait parameters, which best discriminated between THA, TKA, and TAA patients. Self-selected walking velocity has been suggested to be a general indicator of lower-limb function in orthopedic patients [28]. Similarly, SLS has been mentioned as a critical phase of gait in orthopedic patients [29], since during SLS body weight has to be entirely supported by one limb while the other limb swings forward [26]. For this reason, residual impairments in the involved limb (e.g., joint pain and stiffness, reduced joint range of motion, muscle weakness) likely reduced the walking velocity and the ipsilateral SLS phase of our patients. Since the SLS time on one side equals the swing time on the contralateral side, a shorter SLS phase in the involved limb corresponded to a shorter swing phase in the uninvolved limb of our patients. In turn, a shorter swing time was compensated by a longer stance time in the uninvolved limb in order to maintain a nearly symmetrical gait cycle time between the involved and uninvolved limb. In addition, a shorter swing time caused a shorter step length in the uninvolved limb since there was less time to step forward. Therefore, lengthening of stance time and shortening of step length (due to a reduced swing phase) in the contralateral uninvolved limb of TAA and TKA patients are direct consequences of the reduced SLS observed in their involved limb. The differences we observed between THA, TKA, and TAA patients and healthy controls were very similar at normal and fast speeds. However, the difference in walking velocity between TKA patients and controls increased at fast speed, showing that the gait disability of TKA patients was accentuated when they were asked to perform a more demanding task. In contrast, the main effect of side that was observed for some gait parameters at normal speed (mainly SLS), decreased or even disappeared at fast speed. Since SLS represents a critical gait phase [29], this is probably explained by the shorter absolute time that patients had to spend on SLS while walking at fast rather than normal speeds [22].

When considering joint replacement patients separately, our findings mostly agree with those already reported in previous studies. THA patients showed indeed the greatest improvements in walking velocity [8,9], cadence [8], and side-to-side SLS asymmetry [9,10] within the first six months after surgery. Reduced walking speed and shorter step length have already been demonstrated in TKA patients at six months after surgery compared with controls [11]. It is speculated that prolonged gait disabilities demonstrated by patients after TKA could be due to persistent quadriceps muscle weakness in the involved side [30]. Following TKA, patients usually walk with a reduced peak knee flexion angle (i.e., “stiff knee”) during the mid-stance phase of gait [12,13]. This knee flexion avoidance strategy aimed to minimize the use of the quadriceps muscle could have resulted, however, in a shorter SLS in the involved side. TAA patients also demonstrated reduced walking velocity [14,15] and cadence [14] compared to controls at seven months after surgery, as well as shorter step length compared to preoperative values [14]. Brodsky *et al*. showed that SLS did not improve even four years postoperatively compared to preoperative values [6], likely due to persistent reduced sagittal ankle range of motion in the involved side [14-16,31]. In addition, the largely debilitating ipsilateral gait impairments presented by TAA patients induced significant compensatory mechanisms on the contralateral side in order to preserve the main gait functions (i.e., gait progression and stance stability).

It may be speculated that the persistent gait limitations observed in this study for TKA and TAA patients at six months after surgery could be due to the incomplete resolution of the functional deficits before discharge from postoperative physical therapy. It has been shown that clinicians’ and patients’ evaluation of functional recovery can differ at follow-up, with clinicians reporting better functional recovery compared to patients [32]. Clinicians may state that they have no further ability to influence the functional recovery with the available treatment techniques, and believe that time may provide the final step to full recovery. In addition, post-traumatic arthritis as an indication for surgery seems to be a potential factor explaining the between-group differences in gait ability we observed. In fact, the higher the percentage of patients operated on with an indication of post-traumatic arthritis within each group (69% for TAA, 31% for TKA and 0% for THA patients), the greater their gait disability at six months after surgery. However, no significant differences between patients operated with an indication of post-traumatic arthritis and osteoarthritis were detected within each group (data not presented). Therefore, the differences in gait characteristics observed in this study between THA, TKA and TAA patients do not seem to be influenced exclusively by the origin of arthritis. Further research should investigate the between-group differences in gait improvements following surgery also by evaluating the gait ability of patients preoperatively. In addition, self-reported evaluation using questionnaires should be implemented to pre- and postoperative objective evaluation of the patients’ functional recovery.

## Conclusions

Gait disability of patients after unilateral lower-limb joint arthroplasty was more marked for distal than for proximal joint patients at six months after surgery, with a proximal-to-distal progression in the impairment (TAA>TKA>THA). THA patients did not demonstrate significant gait disturbances compared with controls. TAA and TKA patients still demonstrated gait disability compared to controls, with slower walking velocity and reduced SLS in the involved limb. In addition, TAA patients presented marked side-to-side asymmetries in gait characteristics, which represent a potential risk factor for osteoarthritis onset and/or progression in contralateral lower limb joints.

## Competing interest

The authors declare that they have no competing interests.

## Authors’ contributions

NCC, MB, ML, NAM gave substantial contribution to the study conception and design. NCC, JFIG performed data acquisition, and NCC and NAM performed data analysis and interpretation. NCC drafted the article, and all the authors revised it critically for important intellectual content and gave final approval.

## Pre-publication history

The pre-publication history for this paper can be accessed here:

http://www.biomedcentral.com/1471-2474/14/176/prepub
